# Appendicitis epiploicae: An unusual cause of acute abdomen in children

**DOI:** 10.4103/0971-9261.43035

**Published:** 2008

**Authors:** Vipul Gupta, Sunil Kumar

**Affiliations:** Departments of Pediatric and Neonatal Surgery, Ibn Sina Hospital, Kuwait

**Keywords:** Acute abdomen, appendicitis epiploicae, children

## Abstract

Appendicitis epiploicae is described as a rare entity in an 8-year-old boy presented with the features of acute abdomen simulating acute appendicitis. Surgical exploration revealed a torsion of appendices epiploicae of the cecum. The excision of infarcted epiploicae with seromuscular inversion resulted in satisfactory recovery. Authors describe this uncommon cause of pediatric acute abdomen along with the review of pertinent literature.

## INTRODUCTION

Appendicitis epiploicae is an extremely uncommon intraabdominal inflammatory pathology resulting from the infarction of appendices epiploicae secondary to its torsion or thrombosis.[1–3] Most of the disorders of appendices epiploicae are reported in the middle-age group with the involvement of appendages of the transverse and sigmoid colon.[1–3] However, the involvement of cecal appendages remains clinically significant as the clinical presentation mimics acute appendicitis and is rarely diagnosed preoperatively.[1,2] Moreover, appendicitis epiploicae has rarely been described in the pediatric age group with only handful of cases reported in English literature to date.[1–5] Since the clinical presentation is not pathognomic, the diagnosis is rarely performed preoperatively and is often confused with acute appendicitis.[1–3] Thus, we herein report the present case with the aim to spread awareness to the physicians regarding the clinical presentation and management protocol of this unusual cause of pediatric acute abdomen.

## CASE REPORT

An 8-year-old male child presented with 1 day history of the sudden onset of acute pain in the right lower abdomen, which was associated with nausea, few episodes of vomiting and a low-grade fever.

On examination, the child had a temperature of 37.2°C, pulse rate of 110/min and blood pressure of 124/76 mm Hg. Systemic examination revealed no abnormality, except for the presence of tenderness with guarding at McBurney’s point. Digital rectal examination was normal. Laboratory investigations suggested mild leukocytosis with a WBC count of 11000/mm^3^ and 82% polymorphs on differential count. Ultrasound examination of the abdomen suggested minimal free fluid in right iliac fossa with normal solid viscera and the appendix was not visualized.

A provisional diagnosis of acute appendicitis was considered and the patient was taken for surgical intervention. Surgical exploration was performed through Lanz incision, which revealed the presence of approximately 10 ml of serosanguinous fluid in the right iliac fossa with an appendix with normal appearence. Further exploration revealed the presence of gangrenous appendix epiploica on the surface of the cecum. The infracted appendage was excised with seromuscular inversion of the cecal wall and appendectomy. Histopathology revealed a normal appendix with congested, hemorrhagic and partly necrotic adipose tissue suggestive of appendicitis epiploicae. The postoperative period remained uneventful, and the child was discharged after 48 h.

## DISCUSSION

Appendicitis epiploicae is an extremely rare entity in the pediatric age group.[1–3] It is intraabdominal inflammatory pathology of fatty appendages of colon. These appendages are fatty structures with approximately 2-3 cm in diameter scattered over entire colon with covering of peritoneum and are supplied by single artery and vein.[1,3] The infarction of these appendages from either torsion or thrombosis results in inflammatory pathology termed as appendicitis epiploicae.[3] The disorders of appendices epiploicae have a peak incidence in the middle-age group with more common involvement of the appendages of transverse and sigmoid colon.[1–5] However, the involvement of cecal appendages remains clinically significant as it may mimic acute appendicitis.[1,3] Moreover, appendicitis epiploicae remains an extremely uncommon cause of acute abdomen in pediatric age group, which has been scarcely reported and discussed in English literature to date.

The clinical presentation in cases of appendicitis epiploicae is usually atypical and is heralded by the presence of clinical features and signs of peritoneal irritation localized to right side of abdomen. This is mainly indicated by the sudden onset of abdominal pain localized in right iliac fossa with minimal gastrointestinal symptoms in the form of nausea, vomiting, and low-grade fever.[1–4]

The laboratory investigations were found to be inconclusive and usually suggest a normal or slightly elevated leukocyte count.[1–5] Thus, due to the lack of pathognomic clinical features, the diagnosis of this rare entity is rarely considered preoperatively and this condition is most commonly confused with acute appendicitis.

The radiological evaluation in form of abdominal ultrasound examination and CT scan has been found to be useful in preoperative diagnosis in patients with appendicitis epiploicae.[1,6,7] A review of literature suggests that the presence of hyperechoic noncompressible ovoid structure near the colonic wall with the absence of blood flow on vigilant sonographic assessment of the abdomen provides the clue to diagnosis.[1,6] Similarly, CT scan has been found to be useful both for preoperative diagnosis and for further assessment in patients managed conservatively.

The treatment of appendicitis epiploicae is mainly conservative if diagnosed preoperatively.[1,3] Although most of the cases in adults respond to antibiotics and analgesics, the clinical experience with conservative management in pediatric age group is limited due to nonspecific clinical presentation. Therefore, the diagnosis of uncommon intraabdominal inflammatory pathology is usually apparent at the time of surgical exploration undertaken with a provisional diagnosis of acute appendicitis.[1–5] Laparoscopy has been found to be useful both as diagnostic and therapeutic intervention in such cases.[3] The excision of the infracted appendage with seromuscular inversion of cecal wall remains the treatment of choice and results in satisfactory recovery.[2,3]

Thus, appendicitis epiploicae remains an extremely uncommon intraabdominal inflammatory pathology presenting with nonspecific clinical presentation in pediatric age group. We conclude that a high index of clinical suspicion and vigilant radiological assessment may help in providing a clue to diagnosis, and hence, a prompt recovery with conservative management, thereby preventing the morbidity resulting from unnecessary surgical intervention.
